# Special Issue: Recombinant Protein Expression in Microorganisms

**DOI:** 10.3390/microorganisms7090355

**Published:** 2019-09-16

**Authors:** Trygve Brautaset, Svein Valla

**Affiliations:** Department of Biotechnology and Food Sciences, Norwegian University of Science and Technology (NTNU), Sem Selands vei 6/8, 7491 Trondheim, Norway

Microorganisms are widely used in industrial biotechnology as cell factories for the sustainable production of a wide range of compounds and chemicals. For example, virtually any protein from any origin can be efficiently produced using recombinant DNA technology, and bacteria and yeast are, in many instances, the preferred production hosts. Recombinant expression has been a fundamental discipline in molecular biology for decades; yet, we still do not understand some fundamental aspects of this science. Presumably, critical information is hidden in the coding sequences of the genes to be expressed, beyond the well-understood rules concerning rare codons and secondary structure formation at the messenger RNA level. Challenges in recombinant protein expression can occur at any level, including host toxicity, product insolubility and non-functionality, poor translation efficiency, and lack of necessary post-translational modification. Genetic tools and superior hosts have been developed, as well as better software for improved design of expression systems by considering all possible parameters including gene coding sequences, promoters, untranslated leaders regions, transcriptional terminators, mRNA stabilities, protein folding, and translational efficiencies. However, despite of all this progress, recombinant protein expression remains largely a matter of trial and error.

This Special Issue of *Microorganisms* gathers 14 articles addressing various aspects of recombinant protein production, including genetic tools [1,2,3,4,5], differentiation between soluble proteins versus inclusion bodies and protein kinetics [6,7,8], fusion proteins [9], recombinant expression in biofilms [10], continuous cultures [11], and high-cell-density cultivation [12]. The articles cover research conducted in several different microbial hosts, including Gram-negative bacterium *Escherichia coli* [1,2,3,6,7,8,9,10], Gram-positive bacilli [4,5,11], and yeast [11,12,13,14]. Altogether, we think that this Special Issue provides a valuable update on important aspects related to the field recombinant expression in microorganisms.
